# The Influence of Porosity on Fatigue Crack Initiation in Additively Manufactured Titanium Components

**DOI:** 10.1038/s41598-017-06504-5

**Published:** 2017-08-04

**Authors:** S. Tammas-Williams, P. J. Withers, I. Todd, P. B. Prangnell

**Affiliations:** 1 Department of Materials Science and Engineering, University of Sheffield, Sheffield, S1 3JD UK; 2 School of Materials, University of Manchester, Manchester, M13 9PL UK

## Abstract

Without post-manufacture HIPing the fatigue life of electron beam melting (EBM) additively manufactured parts is currently dominated by the presence of porosity, exhibiting large amounts of scatter. Here we have shown that the size and location of these defects is crucial in determining the fatigue life of EBM Ti-6Al-4V samples. X-ray computed tomography has been used to characterise all the pores in fatigue samples prior to testing and to follow the initiation and growth of fatigue cracks. This shows that the initiation stage comprises a large fraction of life (>70%). In these samples the initiating defect was often some way from being the largest (merely within the top 35% of large defects). Using various ranking strategies including a range of parameters, we found that when the proximity to the surface and the pore aspect ratio were included the actual initiating defect was within the top 3% of defects ranked most harmful. This lays the basis for considering how the deposition parameters can be optimised to ensure that the distribution of pores is tailored to the distribution of applied stresses in additively manufactured parts to maximise the fatigue life for a given loading cycle.

## Introduction

Electron beam melting (EBM) is an attractive powder bed based additive manufacturing (AM) technique for the near-net-shape production of high-value titanium components because of the higher build rates compared to equivalent laser based technologies^1^. The AM system developed by Arcam AB, who are currently the only supplier of commercial EBM equipment, employs a rapidly scanned electron beam, focused by electromagnetic lenses, to melt the precursor spread powder layers^1^. Unlike most other AM processes, the entire manufacturing cycle takes place at an elevated temperature, which reduces the residual stress to near zero^2^. Although porosity levels as low as 0.2% can be achieved^1, 3^, the presence of defects is still a major concern for fatigue critical applications, such as those encountered in the aerospace industry^2^.

Tensile test bars machined from Ti-6Al-4V samples built with the Arcam EBM equipment have been typically reported to exhibit yield stresses, tensile strengths, and elongations comparable to those of wrought material^4–7^. In contrast, without post-manufacture hot isostatic pressing (HIPing), the high cycle fatigue life can be significantly lower than for wrought Ti-6Al-4V and exhibit a large degree of scatter^2, 6, 8^ with fatigue lives varying by as much as several orders of magnitude^8^. Following HIPing, fatigue lives have been found to increase while the scatter reduces^9, 10^. Most authors attribute this improvement to the closure of internal porosity, but microstructural coarsening reducing crack growth rates has also been suggested^11^. Recently it was demonstrated that the increase in microstructural size associated with HIPing is of negligible importance compared with the reduction in porosity by applying a heat treatment with the same temperature profile (920 °C for 2 hours) but without the 100 MPa pressure pr﻿ior to fatigue testi﻿ng^9^. Entrained pores mainly originate from trapped gas, but larger defects can also arise when there is a lack of powder fusion owing to poor process control^3, 12, 13^. In addition, the defect population is influenced by the melt strategy employed^3^, component geometry^14, 15^ and post - processing^16, 17^.

The location of the porosity has been shown to be important for fatigue life, with shorter lives associated with cracks originating from pores located near the test piece surface, for Ti-6Al-4V samples produced by both EBM^2, 8^ and selective laser melting (SLM)^18, 19^. Murakami^20^ has suggested that pores generate a stress intensity factor, which is dependent on the size of the defects and is greater for defects at the surface, while finite element (FE) modelling has confirmed that the stress concentration generated by pores is greater when they are within one diameter of a free surface^21^. Inclusions can also result in a much shorter fatigue life when they are located close to a sample surface, in comparison to the bulk^22^. Previously, for aluminium fatigue samples containing pores, it has been shown that it is possible to predict where a fatigue crack will initiate using criteria based on the location of maximum stress and plastic strain concentration^23^. In standard engineering components, where the number density of defects is accurately known, it is possible to predict the likelihood of a defect lying in a location where it will initiate a crack, and so predict the probable lives of components due to defect initiated fatigue^22^. However, for EBM AM, both the pore number density and pores’ effect on fatigue have not been fully characterised.

In this paper we have demonstrated the use of a novel time-lapse X-ray computed tomography (CT) method to improve the understanding of the effect of pores on the fatigue life of EBM components. CT allows the complete defect (pore) distribution in fatigue samples to be identified prior to testing. Only by such a method can the pores that do not lead to fatigue crack initiation be identified and contrasted to the initiating pores on the fracture surface. We have then examined the extent to which we can identify the pores most likely to nucleate a fatigue crack using ranking strategies that include the pore size, aspect ratio, proximity to the surface and to other pores, and the propensity for local plastic strain. This has allowed us to examine the extent to which these factors control which defect is likely to become the initiating one. Furthermore, by combining this knowledge with that regarding the non-uniform spatial distribution of pores in EBM components, general recommendations to optimise the fatigue life of components can be found.

## Results and Discussion

### Conventional Fatigue Test Data

**Figure 1:**
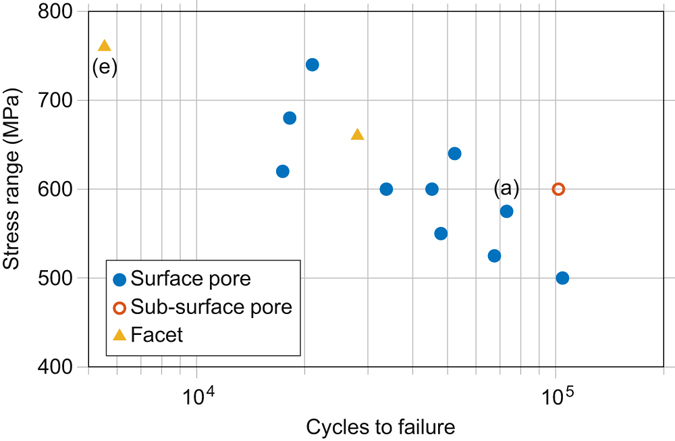
Measured fatigue life of EBM samples. – A S-N curve for uniaxial loading at *R* = 0 showing the number of fatigue cycles to failure against stress range for samples tested in the *z*-direction according to ASTM E466-07. Testing was conducted on cylindrical samples, with a gauge diameter and length of 4.5 mm and 12 mm, respectively, and a blend radius of 9 mm. The marker indicates the feature identified at the crack initiation location for each sample, while the letters enclosed in parentheses indicate the corresponding images in Fig. 2.

The *S-N* curve response of the cylindrical samples tested in the *z*-direction is shown in Fig. 1. Here *z* refers to the build direction (and *x* the rake traverse direction). It is apparent that there is scatter in the data.

Samples were labelled alphanumerically to designate their testing direction and the maximum testing stress. In cases where more than one sample was tested in the same direction and stress level, they were labelled alphabetically in the order of their fatigue life. For example, the three samples tested in the build direction (*z*) with a maximum fatigue stress of 600 MPa are denoted: z-600a, z-600b and z-600c; where z-600a survived the fewest and z-600c the largest number of fatigue cycles.

**Figure 2:**
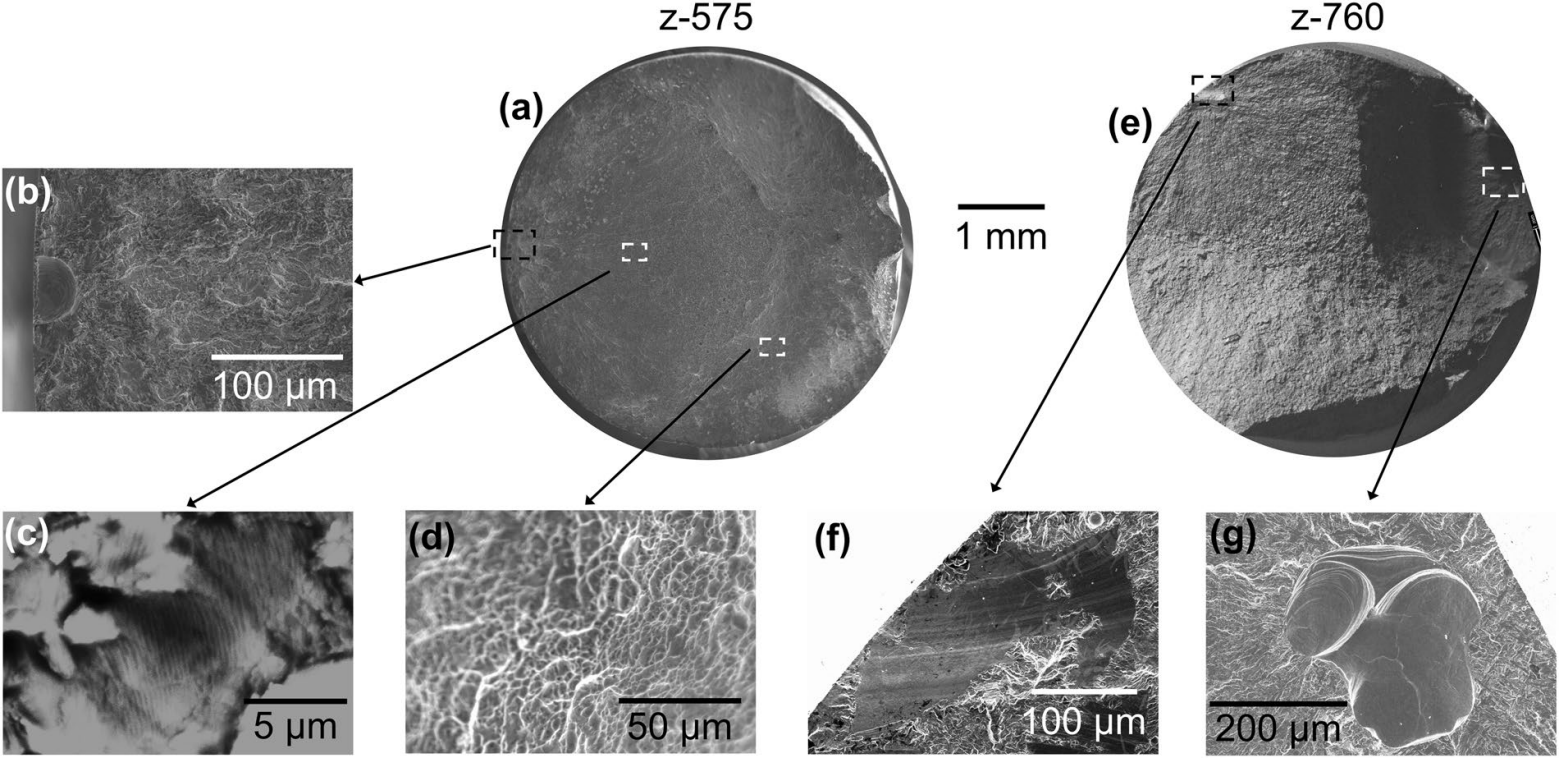
SEM images of example fracture surfaces - (**a**) macrograph of z-575, (**b**) crack initiation from a pore, (**c**) striations in the crack growth region, (**d**) SEM image of the fast fracture over-load region; (**e**) macrograph of z-760. (**f**) Crack initiation from a smooth facet, and (**g**) crack initiation from two conjoined gas pores.

Examples of fatigue fracture surfaces are provided in Fig. 2. Figure 2a–d shows the fracture surface of sample z-575, with clear evidence of a pore at the crack initiation location (Fig. 2b). The crack grew in a plane normal to the direction of loading with only small levels of deviation. Striations are visible at high magnification (Fig. 2c). Once the crack reached a critical size, fast fracture occurred with the final failure region showing typical dimpled overload fracture features^24^ (Fig. 2d).

Sample z-760, shown in Fig. 2e–g, survived the fewest cycles to fracture, having been tested at the highest maximum stress. In Fig. 2e two distinct cracks can be observed that contributed to the failure. The larger of the two cracks initiated at a smooth microstructural facet at the sample surface (Fig. 2f), whereas the smaller crack initiated from an irregular pore near to the sample surface that appears to consist of two partially coalesced gas pores (Fig. 2g).

Fractography of all the samples tested in the *z*-direction revealed that critical fatigue cracks had initiated from facets in 2 samples, pores in 11 samples and, of these, 10 from pores very close to the surface. This result is broadly in line with previous literature regarding the fatigue of EBM Ti-6Al-4V, where fatigue cracks have mostly been found to initiate from pores^1, 2, 8–10, 17, 25^ although one study found facets at the initiation sites^26^. Both samples with facets at the critical crack initiation location were also found to have secondary cracks initiating from a surface pore, as exemplified in Fig. 2e.

Although the major focus of this paper is the nucleation of cracks from pores, and this will be discussed in detail below, the microscopically smooth facets (Fig. 2f) found to be responsible for the initiation of failure in two of the samples are interesting and more typical of fatigue initiation in defect free, conventional wrought specimens where quasi-cleavage facets are commonly observed^27, 28^. Similar smooth facets have also been observed in EBM Ti-6Al-4V by Rafi *et al*.^26^, but they did not suggest they were quasi-cleavage facets. Quasi-cleavage facets typically form in grains or colonies oriented with their basal plane perpendicular to the loading direction with a neighbouring grain/colony favourably orientated for slip^27, 28^. It would require careful analysis of the crystal orientation in the facet location to confirm whether this is the case here, which will be the subject of future research, as the main priority in the current investigation was pore initiated fatigue.

**Figure 3:**
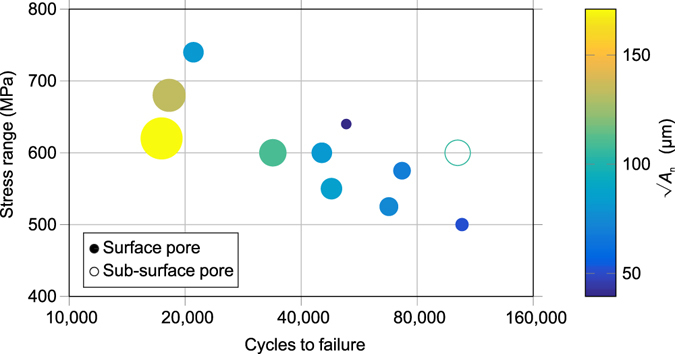
Effect of pore size on fatigue life – S-N curve showing only those samples that failed from porosity for samples tested in the *z*-direction. The size and colour of the markers indicate the size of the pore (*A*_*n*_) measured from SEM images of the fracture surface.

Previous evidence^20, 23^ has suggested that fatigue life is most strongly influenced by the area of a defect normal to the applied stress (*A*_*n*_) rather than its volume. If it is assumed that cracks initiated at the widest cross-sectional area of the pore, then this value can be manually measured on the fracture surface. To reduce measurement error, each pore was measured five times and the mean value calculated. For all samples the standard deviation between measured pore areas was <4%. Figure 3 demonstrates how, in general, larger pores are associated with shorter fatigue lives. Indeed, the shortest life of a sample failing from porosity induced fatigue crack was associated with the largest pore, not the highest stress. By contrast, the relatively large subsurface internal pore appears to have less of a detrimental effect; however, more data is needed to confirm this.

### X-ray CT Imaging

#### X-ray CT Imaging of the Initial Defect Population

**Figure 4:**
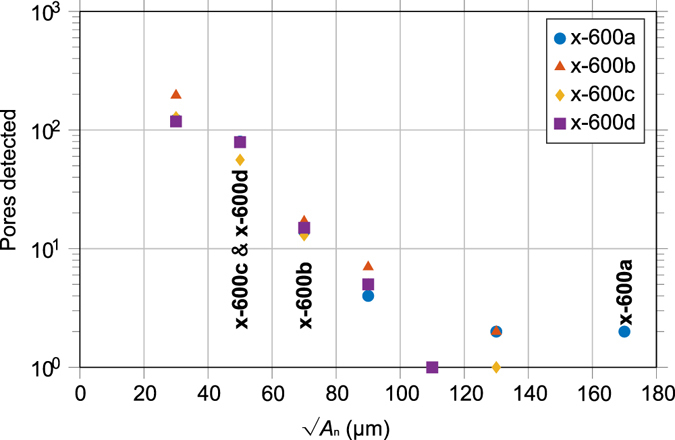
Pore size distribution - Detected by CT in each of the four fatigue samples ~270 mm^3^ gauge volumes prior to testing in the *x*-direction (note log scale). The sample designations (bold) indicate the size of the crack initiating pore for the respective samples.

The standard fatigue data suggests that larger pores are associated with shorter fatigue lives, and fatigue cracks have a tendency to initiate at the surface. However, from a post-mortem analysis of the fracture surface it is not possible to determine whether fracture initiated at the largest pore (or perhaps largest surface pore) or whether there are other factors that contribute to the favourability to initiate cracks. To attempt to answer this, CT has been used to identify and quantify all the pores within an additional four x-600 series samples prior to testing (under uniaxial loading in the rake traverse (*x*) direction with a maximum load of 600 MPa). The pore size distribution in the four specimens is summarised in Fig. 4. While small defects typically dominate the distribution in EBM Ti-6Al-4V^3, 29^, these lie below the resolution limit (~26 μm) of the CT. However, they are unlikely to have a significant effect on the fatigue life. Indeed, it is clear from the initiating defect sizes measured by post-mortem fractography that the initiating defects are significantly larger than the detectability limit (Fig. 2).

#### X-ray CT Imaging of Fatigue Crack Initiation

**Figure 5:**
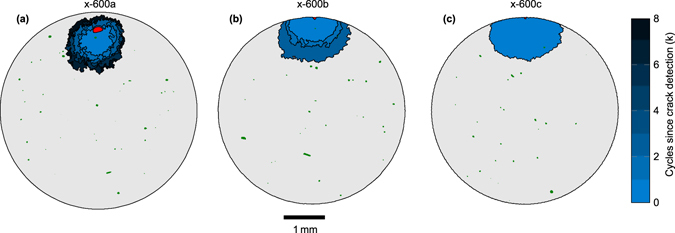
Fatigue crack initiation detected by CT. - Fatigue cracks (blue), the pore that initiated the crack (red), and all other pores (green) within a 1 mm thick slice centred around the plane of the crack, detected by CT after (**a**) 70 k, (**b**) 100 k and (**c**) 120 k cycles. In (**a**) and (**b**) multiple crack tidemarks show the detected crack size every 1 k cycles following first detection of the crack.

By conducting interrupted fatigue testing and periodic CT inspection it was possible to identify both the pore from which cracks initiated, and the number of cycles required to initiate the crack for each of the four samples tested at 600 MPa. Fatigue testing was conducted with identical sample geometry, and to the same standard, as before, but periodic CT inspections of the samples were carried out every 10 k cycles. Cracks were detected by CT after 70 k, 100 k and 120 k cycles in samples x-600a, x-600b and x-600c respectively. No cracks were observed in sample x-600d at 130 k cycles, but it fractured prior to reaching 140 k cycles when the next CT examination was to have taken place. These initiation lives are in line with the initial size of the initiating defect in each case (Fig. 4). Once initiated, crack growth (Fig. 5a and b) does not appear to have been influenced by the neighbouring pores, presumably because their small size and rounded morphology gives rise to relatively little stress concentration compared to that around the crack^30^.

For all four samples monitored, fatigue cracks were found to initiate from pores (shown in red in Fig. 5) the sizes of which are given in Table 1. Sample x-600a cracked at a large internal pore (Fig. 5a), whereas the other samples failed from smaller pores that broke the surface. It is noteworthy that in none of the samples did the crack initiate from the largest measured pore, as is also evident from Fig. 4.

**Table 1:** Details about the pore distributions and the initiating defect in each sample estimated using different ranking strategies.

Sample	Total number of pores detected (>26 μm)	Initiating defect			Rank of initiating defect			
		Location [*d/D*]	Aspect ratio	√*A* *n﻿* (μm)	Ranking 1	Ranking 2	Ranking 3	Ranking 4
x-600a	224	Bulk [2.3]	1.0	170	2	8	2	4
x-600b	298	Surf. [0.70]	1.1	60	23	17	10	10
x-600c	203	Surf. [0.83]	1.0	50	36	9	5	4
x-600d	218	Surf. [0.95]	1.2	50	72	10	7	8
Mean percent of pores ranked as more harmful					14	4	2	2

The smallest crack detected was approximately 0.9 mm (Fig. 5) in length. Since detection of a feature by CT depends on its smallest dimension being approximately twice the voxel size (2 × 10.4 μm), cracks would need an opening displacement >21 μm for reliable identification and so it is unsurprising that the smallest crack observed was significantly longer than the voxel size. The infrequent sampling (every 10 k cycles) also meant that cracks may have grown significantly between inspections. However, as a percentage of total fatigue life, the cycles to initiate a crack detectable by CT was 75–88%, 87–97%, 90–98% and >93%, for samples x-600a, x-600b, x-600c and x-600d respectively. The relatively short propagation life means that fatigue life increases as the number of cycles required to initiate a crack increases.

### Development of a ranking method for initiation site potential

Post-mortem fractography shows that the fatigue life is strongly affected by the size of the initiating defect but CT shows that the initiating defect is rarely simply the largest defect. Here we look at various aspects that might affect the tendency for a pore to initiate a crack.

#### Stress Concentrations around Idealised Pore Geometries

**Figure 6:**
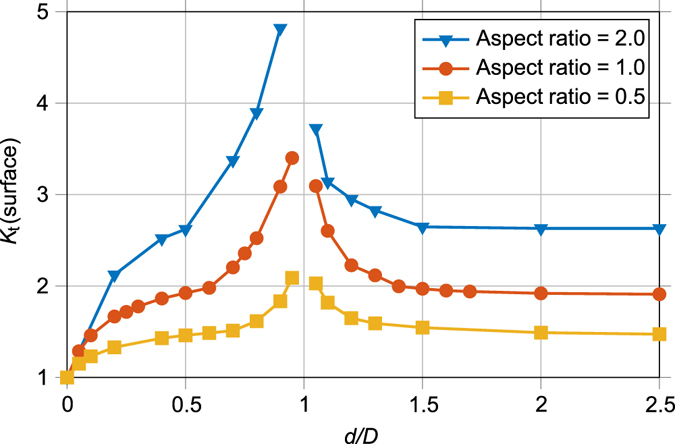
The stress concentration (*K*_*t*_) generated by a pores proximity to a free surface – The maximum increase in stress predicted by linear elastic FE modelling of pores at various depths (*d*) from the surface, normalised by the pore diameter (*D*) normal to the loading vector. Results are shown for idealised spheres/oblate spheroids with three different aspect ratios.

**Proximity to the surface:** Our limited evidence suggests that surface or near surface defects are more detrimental to fatigue life than internal ones. This could be because of the increased stress concentration associated with pores near the surface^21^. It has been found previously that approximately 97% of defects within Ti-6Al-4V samples manufactured via EBM are nearly spherical (aspect ratio < 1.5) gas pores^3^. Spheres/spheroids have thus been used as idealised geometries to estimate the effect of pores near a free surface on the local elastic stress concentration (*K*_*t*_) for three different aspect ratios. For these results to be applicable irrespective of void size, the pore location has been made dimensionless by dividing the maximum depth from the surface (*d*) by the pore diameter (*D*). Values < 1 signify that the pore that is open at the surface; >1 that the pore is completely enclosed. From the results of this FE analysis shown in Fig. 6, it can be noted that there is a large increase in *K*_*t*_ when the pore is close to touching the surface, regardless of the aspect ratio of the void. In fact, in a purely elastic condition, when *d/D* = 1 the pore would be just touching the surface and the infinitely small volume of the surface ligament would lead to an infinite stress concentration. Therefore, *d/D* = 1 is an asymptote of the stress concentration curve. Conversely, when a pore is further than one diameter from the free surface (i.e. *d/D* > 2) the effect on *K*_*t*_ becomes negligible.

Both fractography and CT analysis indicate that all the surface pores that led to critical fatigue cracks lay within a diameter of the surface where the rise in *K*_*t*_ is most significant. This suggests that the increased stress concentration near a surface is an important contributor to the propensity of fatigue cracks to initiate at surface pores.

It should be noted that we have not considered any effect of conducting the fatigue testing in ambient, non-inert, air. Recently, when Serrano-Munoz *et al*.^31^ fatigue tested aluminium samples in both ambient conditions and a vacuum chamber, they found that the detrimental effect of surface pores was removed by testing under a vacuum. Thus, a non-inert atmosphere may have also contributed to the tendency for critical fatigue cracks to initiate at the sample surface.

**Figure 7:**
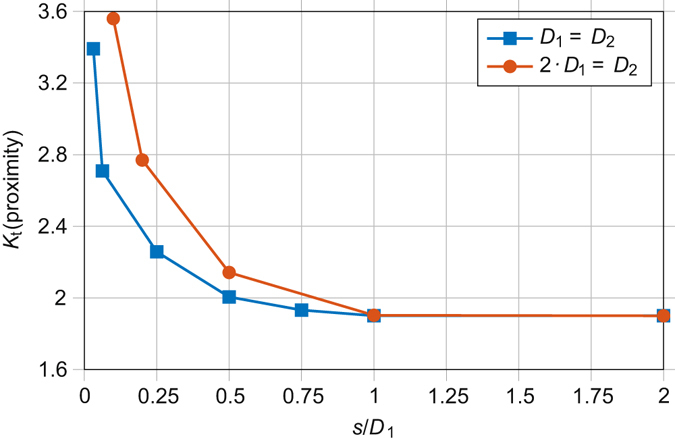
The stress concentration (*K*_*t*_) resulting from pores in close proximity to one another – The maximum increase in stress predicted by linear elastic FE modelling of two idealised spherical pores in close proximity. Loading is normal to the vector between pore centres. The separation (*s*), i.e. solid material, between pore edges has been normalised by the diameter (*D*_1_) of one of the spheres. Results are shown for spheres of equal diameters (*D*_1_ = *D*_2_) and one sphere with twice the diameter of the other (2·*D*_1_ = *D*_2_).

**Proximity to other defects:** Another aspect that could lead to increased *K*_*t*_ and thus encourage crack nucleation is when two pores are in close proximity. The stress concentrating effect of two pores lying in a plane normal to the direction of loading on the *K*_t_, is shown in Fig. 7. Again, the locations have been made dimensionless, this time by dividing the separation (i.e. solid material) between the pores (*s*) by the one of the pore diameters (*D*_*1*_). Results are shown for two identical pores interacting with each other (*D*_*1*_ = *D*_*2*_) and for one pore with twice the diameter of the other (*D*_*2*_ = 2∙*D*_*1*_). The increase in *K*_*t*_ is only noticeable when the pores are separated by less than one diameter and only significant when closer than half a diameter. The results vary slightly when the pores have different sizes, but the overall trends are similar. Given the relatively low number density of pores observed in our case by CT, it is unsurprising that the fracture surface analysis and CT examinations revealed no cases of crack initiation at pairs or clusters of pores. Of course, it is impossible to disregard the possibility that smaller pores, below the resolution limit of the CT, did exist close to the initiating pore and contribute to the stress concentration. Similarly, pores away from the crack plane may also have contributed to fatigue crack initiation in the samples not subjected to CT, with this possibility remaining untested in present work.

**Figure 8:**
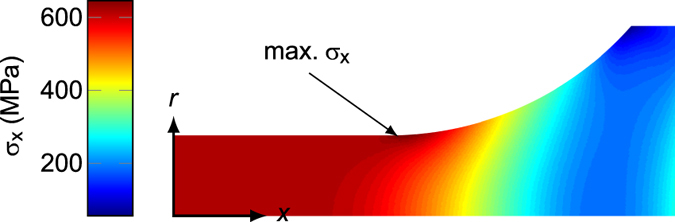
Tensile stress (*σ*_*x*_) distribution within a homogenous fatigue test piece – The axisymmetric tensile stress predicted by linear elastic FE modelling in the *x* and *r* directions, where *x* and *r* are the distance from the sample centre, aligned with, and normal to, the axis of symmetry, respectively.

**Non-uniform stress field**: Finally, the tensile stress distribution expected within a homogeneous fatigue test sample loaded to 600 MPa is shown in Fig. 8. Despite the large blend radius from the reduced gauge section to the gripped end, the stress distribution in the fatigue test samples is somewhat non-uniform, with a 5% higher stress predicted near the sample neck in comparison to the gauge length. A similar effect of the sample geometry was noted by Li *et al*.^23^, and was found to contribute to the stress/strain concentration at the pore that led to the fatal fatigue crack. However, given that a spherical void results in a *K*_*t*_ > 2, and can potentially be much larger if the void is near the surface, it is unsurprising that none of the initiating pores were located in regions where the sample geometry would have (slightly) increased the stress.

#### Ranking the harmfulness of the pores based on time-lapse CT

To quantitatively evaluate the factors that are important in determining the fatigue life, a number of methods of ranking the potential harmfulness of each of the defects identified by CT were applied:

**Ranking 1: Defect size**. Simply assumes the potency of a defect is related to size (maximum area normal to the stress, *A*_*n*_) measured by CT prior to testing.

**Ranking 2: Murakami’s equation**. Murakami^20^ has shown that defects such as pores are equivalent to cracks of a similar stress intensity from the viewpoint of fatigue. The maximum stress intensity factor (*K*_*max*_) generated by an internal defect is given by:

1 $${K}_{max}=0.5\cdot {\sigma }_{{\rm{\infty }}}\cdot \sqrt{\pi \sqrt{{A}_{n}}}$$

where *σ*_*∞*_ and *A*_*n*_ are the global applied stress and the pore area normal to the applied stress respectively. According to the analysis of Murakami^20^, when a defect is at a surface, the constant (0.5) in equation (1) is increased to 0.65 to reflect the greater stress intensity arising from surface cracks. Thus, an approximation of the stress intensity factor range (*ΔK*_*I*_ = *K*_*max*_ & *σ*_∞_ = *Δ* *σ*, since *R* = 0) for all the pores detected by CT was calculated and ranked, with an allowance for the apparently greater potential for surface pores to initiate cracks.

**Ranking 3: Defect size**, **location**, **aspect ratio and proximity to other pores and the surface**. Here we rank the pores from the CT data according to the approximate elastic tensile stress concentration. This ranking includes the stress concentration from proximity to a surface *K*_*t*_(surface), the effect of pore aspect ratio *K*_*t*_(AR) (Fig. 6), proximity to other pores *K*_*t*_(proximity) (Fig. 7) and the non-uniform stress *σ*_*x*_(position) (Fig. 8) across the sample:

2 $${\rm{\Delta }}{K}_{I}({\rm{r}}{\rm{e}}{\rm{l}}{\rm{a}}{\rm{t}}{\rm{i}}{\rm{v}}{\rm{e}})={\sigma }_{x}({\rm{p}}{\rm{o}}{\rm{s}}{\rm{i}}{\rm{t}}{\rm{i}}{\rm{o}}{\rm{n}})\cdot {K}_{t}({\rm{s}}{\rm{u}}{\rm{r}}{\rm{f}}{\rm{a}}{\rm{c}}{\rm{e}})\cdot {K}_{t}({\rm{A}}{\rm{R}})\cdot {K}_{t}({\rm{p}}{\rm{r}}{\rm{o}}{\rm{x}}{\rm{i}}{\rm{m}}{\rm{i}}{\rm{t}}{\rm{y}})\cdot \sqrt[4]{{A}_{n}}$$

An algorithm written in MATLAB was used to analyse all the pores detected by CT to determine each pores normal area, radial location and dimensionless values for aspect ratio, *d/D* and *s/D*. It was assumed all pores had a smooth spheroidal morphology. The radial location was used to estimate the stress due to the sample geometry (Fig. 8). The dimensionless values were then combined with the results of the FE modelling shown in Figs 6 and 7 to assign each pore an elastic tensile stress concentration value. By multiplying this with the fourth root of the normal area, a relative stress intensity factor was calculated. The methodology is described in more detail in the supplementary information. The empirical nature of equation (2) allows the algorithm to run in orders of magnitude less time than required to mesh and model all the pores within a microstructurally faithful FE model. Clearly, the expression in equation (2) is not the true stress intensity factor for each pore, however it can be used to rank the relative harmfulness of each pore.

**Ranking 4: Plastic stress-strain concentration**. Cracks were found by Li *et al*.^23^ to initiate in aluminium samples at the location of maximum plastic stress-strain concentration (*k*_*g*_). To evaluate this requires FE modelling of the fatigue test piece and porosity to locate and calculate the location of greatest *k*_*g*_, given by:

3 $${k}_{g}=\sqrt{{K}_{\sigma }\cdot {K}_{\varepsilon }}$$

where *K*_σ_ and *K*_*ε*_ are the local principal stress and plastic strain concentrations, respectively. While FE meshes can be generated from CT data, the number of pores meant that it was not feasible to individually mesh and model them all, and the computational cost of modelling the entire fatigue gauge length with a fine enough mesh to retain accuracy was prohibitive. Therefore, only those pores judged to be among the 5% most detrimental by method 3 described above were modelled. Sub-volumes around the selected pores were extracted, with a size 5 times the pore diameter and taking into account any other pores or sample surface as originally defined by the CT data. The loading conditions applied to the models were defined by their location within the sample and the stress distribution shown in Fig. 8.

Thus, four methods with varying levels of complexity were used to rank the pores potential relative detrimental effects. In Table 1, the ranking of the pore at which the crack actually initiated by each of the methods outlined above is given. All three ranking methods that take into account the local environment outperformed that based on size alone (ranking 1) confirming the importance of local environment and in particular the importance of proximity to the surface. Overall the best performing are ranking methods 3 and 4, which given the complexity of 4 suggest that it is sufficient simply to take into account the elastic stress concentration arising from proximity to the surface and the neighbouring pores. It is noteworthy that none of the methods applied were able to correctly predict the actual initiating defect, highlighting the stochastic nature of fatigue.

While computational limitations preclude using a full model including all the defects, the initiating defect was always within the shortlist comprising the 5% of pores highlighted as likely to be most detrimental by ranking 3. Similarly, the neglect of pores having diameters smaller than the CT resolution (<26 μm) does not seem to be important for fatigue crack initiation in this case. Instead, cracks always initiated at a pore of relatively high *ΔK*_*I*_. Consequently, if the maximum *ΔK*_*I*_ in a sample could be reduced by better control of the largest defect size (particularly near the surface), the fatigue life would also likely increase.

More complex FE modelling of the pores did not result in a significantly more accurate identification of the likelihood of a pore being the crack initiation site. This is in contrast to the work of Li *et al*.^23^ who found fatigue crack initiation in aluminium samples occurred at the region of the highest plastic stress-strain concentration calculated with an FE model generated from CT data. This failure to predict the actual defect initiating a crack could be due to a number of factors, including errors in segmentation of the CT data and the lack of any consideration of the local microstructure. Furthermore, the resolution of the CT may have been insufficient to detect the true geometry of the porosity, whereas the pores analysed by Li *et al*.^23^ were significantly larger than the voxel size; hence, they would have been able to extract a more accurate representation of the pore.

Since FE modelling of the imaged pores was unable to identify the initiating pore, it suggests that we must either use higher resolution CT to get a more accurate model of the stress distribution or account for effects other than stress. For example, the propensity of surface pores to initiate cracks may have been amplified by the testing taking place in a non-inert atmosphere. However, without carrying out multiple extra tests in an inert atmosphere it is impossible to define how significant this effect is.

**Figure 9:**
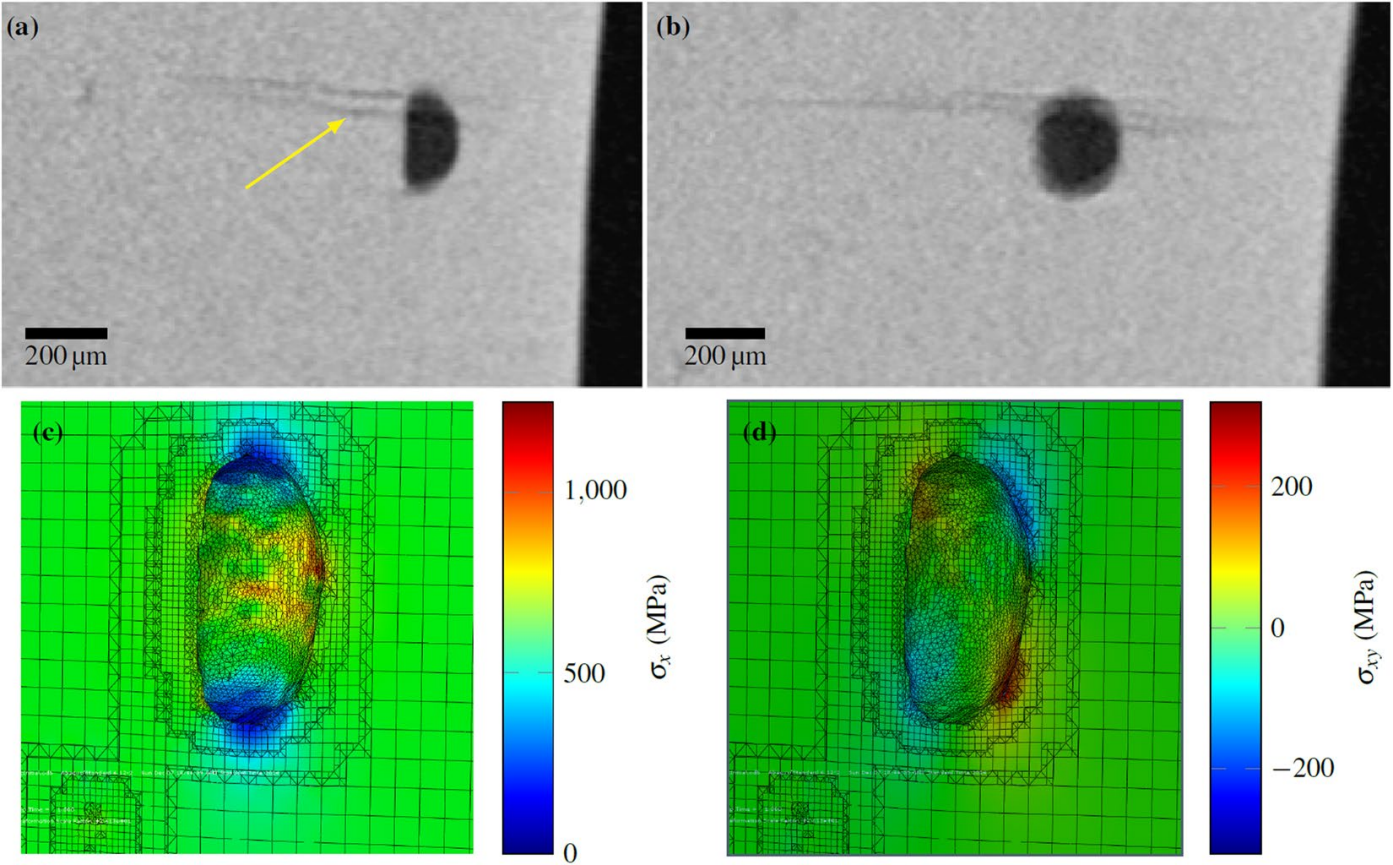
Fatigue crack initiation location in sample x-600a - Slices of the CT data in: (**a**) *x-y* plane and (**b**) *x-z* plane. Here, *x* is both the raking direction during the EBM build and loading direction during fatigue testing, while *z* is the build direction. Results of the analysis of the pore geometry detected by CT prior to testing with half the model visible, showing (**c**) tensile stress and (**d**) shear stress.

By word of caution it is noteworthy that the crack initiating from the large interior pore in sample x-600a (arrowed in Fig. 9a) appears not to have nucleated at the mid-riff where the tensile stress concentration (Fig. 9c) is greatest, but near the pole. Given that the shear stress (Fig. 9d) is large near here this could indicate that in this case the fatigue crack initiation and early growth is dominated by shear within persistent slip bands^32^. Alternatively, it could be simply an indicator of the stochastic nature of fatigue crack initiation associated with local microstructural factors. Unfortunately, the resolution of the CT data was not high enough to confirm whether this was the case in the other samples, where the pores at the crack initiation site were much smaller.

### Importance for Manufacturing Strategy

**Figure 10:**
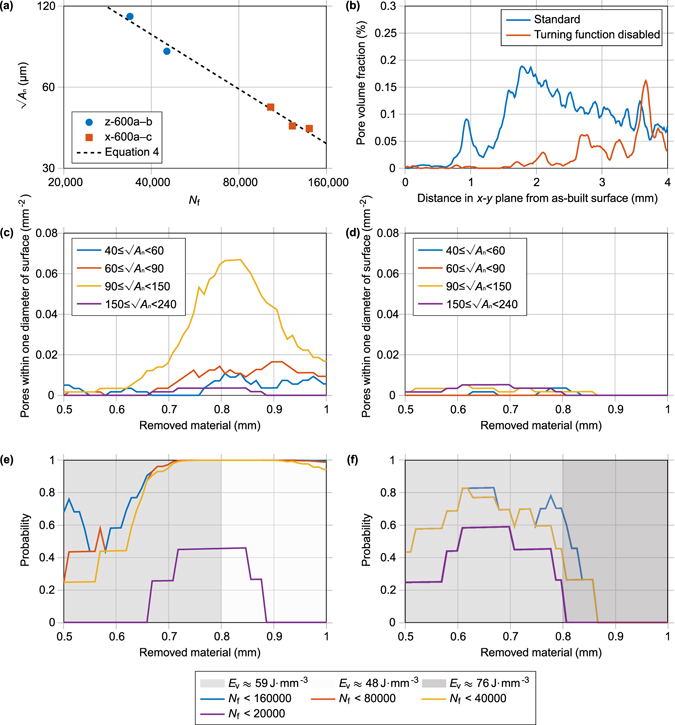
Distribution of porosity near the as-built surface of EBM components and its possible effect on fatigue life – (**a**) the size of pores identified on the fracture surface by SEM for samples against cycle to failure for samples tested at 600 MPa and failing from surface pores. (The standard deviation in measured pore size is contained within the marker size). (**b**) The total volume fraction of porosity with distance from the surface for standard and modified melt strategies (adapted from ref. 3). The number density of pores within one diameter of the surface when machining to different depths for a sample melted with (**c**) standard, and (**d**) slightly modified melt strategies. The probability of at least one pore with a size large enough to cause failure within a given number of cycles appearing near the machined surface for a (**e**) standard sample and (**f**) modified sample. In (**e**) and (**f**) the energy density (*Ev*) used to melt each area is given by the background colour.

It has been shown that fatigue cracks tend to initiate at locations of high relative stress intensity, particularly at pores brought to the surface during machining. From Fig. 2 it is apparent there is a relationship between pore size at the crack initiation and the total sample life (*Nf*). This is demonstrated more clearly in Fig. 10a, where the relationship (equation (4) has been estimated by fitting a straight line (by simple linear regression, *R* ^2^ = 0.99) to the logarithmic data regarding samples tested at 600 MPa that failed from surface pores. The values in parentheses denote the standard error of the fit.

4 $$\sqrt{{A}_{n}}={10}^{5.1(\pm 0.2)}\cdot {{N}_{f}}^{-0.67(\pm 0.04)}$$

Samples tested in both the *x* and *z* direction appeared to follow the same trend. Whilst an inverse logarithmic relationship between pore size and sample life is hardly surprising, it is of note because, unlike some other manufacturing process, in EBM the strategy can be changed to alter the distribution of defects, for example moving them away from the surface to reduce their stress concentration/intensity. In addition, it is possible to estimate the pore size associated with various fatigue live. Thus, for a sample to withstand 10,000, 20,000, 40,000, 80,000 and 160,000 cycles prior to fracture the initiating pores size (*√A*_*n*_) is calculated to be less than approximately 240, 150, 90, 60 and 40 µm respectively. Of course, with the limited data available, the error in the estimated pore sizes is very large and the values should be applied with caution. With more data the error would likely reduce and allow the pore size to be better linked to fatigue life. Nonetheless, we can use this data as a demonstrator to examine the effect of pore size on fatigue life; more data may change the absolute allowable pore size but the overall trends would almost certainly remain the same.

In standard samples manufactured by EBM, there is a tendency for a greater volume fraction of pores to be found near the surface of a component. An example of the variation in the as-built pore volume fraction with depth from the vertical surfaces of a 10 × 10 × 25 mm^3^ cuboid sample manufactured with the standard EBM melt strategy is given in Fig. 10b (data taken from a separate study^3^). Two peaks are visible, caused by the reversal of the beam transverse direction and the increase in speed that accompanies the reversal, and are discussed in detail elsewhere^3^. The very edge of the sample, melted by the contour strategy, has a relatively low volume fraction and number density of defects.

Unfortunately, the surface finish of EBM components is currently fairly poor^6^, so it is likely that some level of surface machining will be required before putting components into service. This could move the pores responsible for the peaks in pore volume fraction identified in Fig. 10b close enough to the surface of components to result in an increase in their stress concentration factor. The CT data from the standard cuboid (Fig. 10b) was therefore interrogated to calculate how many pores would be brought to a detrimental location with different levels of material removal. Between 0.5 mm and 1 mm of material was removed from the virtual model of the as-built sample (likely to be similar that removed in an industrial application) and the number of pores brought within one diameter of the surface calculated. From the calculated number of pores, shown in Fig. 10c binned by size, it is clear that the level of material removal can significantly affect the number of pores present per unit area of machined surface, with a peak at around 0.8 mm, before dropping with further material removal.

It should be noted that the fatigue samples analysed in this paper were much smaller than the as-built specimen size, leading to around 6 to 8 mm of material being removed during machining. From Fig. 10b, it can be seen that not only does this remove the initial peaks, but that there is a gradual decline in pore volume fraction further from the surface. These fatigue samples may have exhibited relatively high fatigue life in comparison to components built to near net shape, as they contain a lower fraction of porosity. The effect of this surface region containing a greater number and volume of pores should be considered when designing near net shape AM components for fatigue loading.

With a distribution of pore sizes, the probability of a pore appearing close enough to the surface of a fatigue sample to have a significantly raised stress level with different levels of machining can also be approximated. The Poisson distribution provides a simple approximation of the probability (*P*) of at least one defect appearing^22^:

5 $${P}=1-{\text{e}}^{-\lambda }$$

where *λ* is the average number of pores, i.e. the number density presented in Fig. 10b multiplied by the surface area of the fatigue specimen. Using equation (5), a probability plot of the fatigue life of samples with different machining depths can be generated by assuming that fatigue cracks will initiate at the largest surface pore. From the analysis of the standard sample shown in Fig. 10e, it can be seen that changing the machining depth from 0.55 to 0.75 mm changes median expected life from approximately <160,000 to <20,000. While this elementary analysis (with uncertainties in the exact correlation of pore size to fatigue life) is not rigorous enough to provide accurate indications of the fatigue life of components, it does highlight how small changes to the depth of removed material can make large changes to the pore distribution, and thus probable fatigue life. With more detailed statistical data regarding both pore size distributions and the number of cycles expected, this may be a stepping stone towards predicting the fatigue life of components built to near net shape by EBM.

Perhaps a more useful application of this analysis lies in demonstrating the potential improvement in fatigue life achievable by altering the melt strategy to avoid defects appearing near the sample surface. If the hatching turning function is disabled (a discrete option in the control software) the beam provides a greater line energy near the surface of components by not increasing its speed when starting a new hatch line, which locally increases the size of the melt pool and decreases the likelihood of pores appearing^3^. In a sample produced with this adjustment no pores greater than 40 µm were observed between 0.9 mm and 1 mm from the surface (Fig. 10d). If a fatigue sample were tested with this modification and between 0.9 mm and 1 mm removed, it would be expected that no surface pore initiated fatigue cracks would lead to failure before 160,000 cycles (Fig. 10f). In this case, sub-surface pores or quasi-cleavage facets may become the primary initiation modes.

Increasing the energy input is not without drawbacks, in addition to increased energy costs, it is associated with a greater microstructural size and reduced material hardness^29^, which may have unintended consequences on the fatigue life. However, when a heat treatment was applied to EBM samples to increase microstructural size, the difference in the fatigue life was negligible in comparison to as-built material^33^. Moreover, the improvement in fatigue life following HIPing suggests that a larger microstructural size and fewer pores results in improved fatigue performance. Nonetheless, care must also be taken to avoid overheating the edge of parts, which can lead to them “peeling up” and poor control of the component geometry^34^. Furthermore, the larger melt pools generated by this modification resulted in more large pores than seen in the standard samples within 0.8 mm of the surface, which means that with a smaller machining depth than this, this sample would likely have a shorter fatigue life than a standard sample. Thus, the importance of picking a suitable machining depth for different melt strategies and energy densities has been highlighted.

For engineering components, the geometry would influence both the stress distribution and the volume fraction of porosity^14^. Hence, CT and stress analysis of a first iteration component could be used to identify the most damaging pores, before alteration of the melt strategy to avoid their appearance. For example, the energy density could be increased in specific regions likely to experience high stress to ensure minimal defects, while only leading to a slight increase in the energy cost of the build.

## Conclusions

This paper confirms that the fatigue lives of samples manufactured by EBM AM are strongly influenced by the presence of retained porosity, although crack initiation at quasi-cleavage facets can also arise on occasion. We have also demonstrated the benefits of using time-lapse X-ray CT to identify the defect population prior to fatigue testing. Conventionally one focuses on the initiating defect, located on the fracture surface and indeed larger defects were typically associated with shorter fatigue lives. Non-destructive time-lapse imaging reveals, however, that the initiating defect is rarely the one that the approach of Murakami^15^ would indicate as being the most deleterious defect. Indeed, in many cases a defect at the surface, which would be considered more benign from a conventional fracture mechanics viewpoint, will often initiate a crack much earlier than the one suggested the Murakami analysis. This tendency for defects at the surface to be more serious in terms of fatigue life than conventional fracture mechanics would suggest is important in understanding and improving the fatigue life of AM components where such surface defects can be avoided by careful control of manufacturing process conditions. On the other hand, with the limited number of CT results presented here, one must be cautious to avoid over emphasising these results; more research is required in this area to corroborate our findings.

The non-uniform spatial distribution of porosity in EBM means that different surface machining operations will lead to different levels of porosity at the machined surface and thus is likely to result in different fatigue lives. By altering the melt strategy and machining depth, it may be possible to optimise the fatigue life by avoiding defects near the surface. This will enable materials scientists and engineers to develop probabilistic models for component life prediction in AM parts - thinking particularly on those components that are designed to exploit AM, e.g. structurally optimised. While it is not possible to conduct X-ray CT on every manufactured article, it could be used along with FE modelling and our stress concentration approach to identify regions where there is a combination of high stress and a propensity for defect formation and thereby to help tailor the deposition scan strategy and process parameters to extend fatigue life.

## Method

### Sample Manufacture

The fatigue samples were machined from additively manufactured material built at the University of Sheffield on an Arcam S12 EBM machine, using pre-alloyed gas atomised Ti-6Al-4V powder feedstock. Powder was deposited in 70 μm layers in the *x-y* plane with a rake moving in the *x-*direction before the electron beam was used to preheat then melt the powder where required. Standard settings were used and these are described in detail elsewhere^3, 34^. Chemical analysis of the powder gave the following weight fractions (%), Al: 6.4, V: 4.2, C: 0.009, Fe: 0.16, O: 0.126, N: 0.013 & H: 0.001. An initial build was undertaken to manufacture cylindrical samples of diameter 16 mm and height 60 mm, aligned with the build direction (*z*). The remaining powder was used to conduct a second build to manufacture 16 mm square cross section samples of the same 60 mm, lying in the build plane and aligned with their long direction parallel with the rake travel direction (*x*). Some differences have been observed between in the pore distribution in samples manufactured in different orientations. The effects are likely to be most significant close to the surface where the melt pool size is affected by both the control software altering the electron beam parameters, and the reduced conductivity of the surrounding powder in comparison to the solid material^35^. The affected surface region was subsequently removed by machining prior to testing. All hatch lengths were kept below the 100 mm length recommended by Arcam to keep a consistent melt pool size and defect population. Cylindrical fatigue test pieces, with a gauge diameter and length of 4.5 mm and 12 mm, respectively, and a blend radius of 9 mm, were machined from these two sample sets with their fatigue loading axis parallel to the build (*z*) and raking direction (*x*), respectively.

### Standard Fatigue Testing

Load controlled uniaxial fatigue testing was carried out in accordance with ASTM E466-07 on the samples orientated in the *z*-direction. An *R*-ratio (minimum to maximum load) of 0 was applied during testing at a frequency of 0.25 Hz using a trapezoidal loading waveform. Samples were tested to fracture. Fracture surface analysis of the failed fatigue samples was carried out using a CamScan FEG-SEM. Pore sizes on the fracture surfaces were quantified using the ImageJ image analysis software^36^.

### Interrupted Fatigue Testing and CT Analysis

The samples tested in the *x*-direction were scanned by X-ray CT as-machined to characterise the pores within the sample gauge volume prior to testing at the Henry Moseley X-ray Imaging Facility at the University of Manchester. Interrupted fatigue testing was then conducted to the same standard as described above with a maximum stress of 600 MPa. The tests were interrupted and CT scans undertaken every 10k cycles. The Nikon Metrology 225/320 kV Custom Bay machine was used to scan a 16.6 mm length of the sample centred on the gauge length. Scanning was performed with a 95 kV accelerating voltage, a 130 μA current and a molybdenum reflection target. 3142 projections were acquired with a one second exposure time giving a total scan time of approximately 54 minutes. 3D data was reconstructed from the 2D radiographs using a filtered back projection algorithm. When scanning by CT, a compromise was struck between spatial resolution and field of view. A voxel size of 10.4 μm was selected allowing the whole of the gauge volume of the fatigue specimens to be imaged. At this resolution pores with an equivalent diameter (diameter of a sphere of the same volume) greater than 26 μm can be reliably detected. Further discussions of the factors influencing defect detection by CT are available elsewhere^37, 38^. The CT data was segmented into a binary volume (solid/void) using the automatic Otsu method^39^ to decide a threshold in Avizo, then MATLAB was used to fit a convex hull to the cylindrical fatigue test pieces to identify any surface breaking porosity. More details are available in the supplementary information and Supplementary Fig. S1, and Tammas-Williams *et al*.^30^. MATLAB was also used to quantify the pore sizes and locations.

During CT scanning a tensile rig was used to apply 600 MPa tensile stress in order to hold open any cracks to aid their detection. As a result, these samples experienced an approximately 1-hour dwell loading cycle every 10,000 cycles. However, for Ti-6Al-4V this low frequency of dwell loading is unlikely to have significantly influenced the results^40^. Once a crack was detected, for samples x-600a and x-600b the number of cycles between inspections was reduced to 1000 to allow a qualitative inspection of the 3D crack growth.

### Finite Element Modelling

FE modelling of the effect of voids on the local stress distribution was carried out using Abaqus Standard 6.14. Ideal elastic models of generic pore geometries, based on oblate spheroid voids, were created within the Abaqus CAE software, using 3D stress elements with a size 0.05 that of the diameter of the void under investigation, to systematically investigate the effect of pore aspect ratio, and proximity to a free surface, or other pores, on the local elastic stress concentration. A Poisson’s ratio of 0.32 was used throughout. Symmetry of the model geometry and loading conditions meant that only a quarter of each pore was required to be modelled. Axisymmetric FE modelling was also carried out to examine how the stress distribution was influenced by the fatigue sample geometry. The stress distribution around selected real pores, whose actual shape was characterized by X-ray CT, was also studied in FE models. To convert the 3D CT data into meshes for use with FE modelling, the segmented data was imported into the ScanIP 7.0 software, which automatically generates a mixed tetragonal and hexahedral 3D stress mesh with an element size based on the voxel size, and element refinement around, and decimation away from, the pore surfaces^41^. Abaqus CAE was then used to import the file from ScanIP, apply material properties and loading and boundary conditions. Idealised elastic-plastic modelling (with a Young’s Modulus of 120 GPa and a yield stress of 1 GPa, followed by perfectly plastic deformation) was conducted.

## Electronic supplementary material

Suppelmentary Information
