# Supplementary material for: The Influence of Porosity on Fatigue Crack Initiation in Additively Manufactured Titanium Components
Source: Sci Rep. 2017 Aug 4;7:7308. doi: 10.1038/s41598-017-06504-5 (PMC5544733; doi:10.1038/s41598-017-06504-5)
Supplement: Supplementary file 1 — Suppelmentary Information [file 41598_2017_6504_MOESM1_ESM.pdf]

The Influence of Porosity on Fatigue Crack Initiation in Additively Manufactured Titanium Components

Supplementary Information

Samuel Tammas-Williams[1,2], Philip J Withers[2], Iain Todd[1], Philip B Prangnell[2]

[1] Department of Materials Science and Engineering, University of Sheffield, Sheffield S1 3JD, UK

[2] School of Materials, University of Manchester, Manchester, M13 9PL, UK

Identifying and Ranking Porosity from X-ray CT Data

All segmentation was first conducted in Avizo, as detailed in the experimental methodology section, and a stack of binary images was imported into MATLAB. An example of a slice of the 3D reconstructed data from sample x-600c is shown in Figure S1a alongside the same slice after segmentation by the Otsu method (Figure S1b).

The first step of the script was to recreate the intended fatigue bar geometry and identify the open porosity that was missed by segmentation in Avizo. This was achieved by analysing each slice individually and fitting a convex hull to the data. An example of a slice and the corresponding convex hull is shown in Figure S1b and c respectively. A convex hull is the smallest convex geometry that still contains all the solid material. In practice, this will draw a straight line across the open porosity (arrowed) as shown in Figure S1c. By masking the ideal shape with the original data it was then possible to identify all the porosity, including pores open to the surface which were missed by the standard techniques in Avizo. The pores identified in Figure S1d include the open pore (arrowed) that actually led to the fatal fatigue crack in sample x-600c. The linear segments used to construct the convex hull meant that the size of the open porosity was slightly underestimated, but this effect is very small. The idealised 'hull' shape was also used to define a radius and center of the solid material for each slice.

The individual pores were then separated and their centroids calculated using in-built MATLAB functions. It was assumed that the fatigue samples' loading direction, x, was perfectly aligned with the rotational axis during CT scanning, and thus the area of the pore normal to the loading direction (A_n) was simply the area of the pore in the y-z plane. This normal area was also used to provide the equivalent diameter of a spherical void of the same normal area (D_n).

The aspect ratio of all the internal pores was calculated using their maximum dimension in the y-z plane divided by their height in x. It was assumed all the pores were oblate spheroids and this was used to assign a stress concentration due to the pore aspect ratio (K_t (AR)), based on the FE modelling results shown in Figure 6. The aspect ratio of pores open to the surface was calculated based on the aspect ratio of the 2D opening in the sample surface.

The idealised hull geometry (Figure S1c) was also used to create a distance map for each slice, which denoted the distance of each internal voxel from the ideal shape. In this way, the depth of the open porosity, in addition to the distance of all the internal porosity from the surface, could be calculated. The maximum and minimum depth of each pore was then calculated by checking the distance map value at all the voxels around each pore perimeter. Those pores with a minimum depth of one voxel (i.e. touching the idealised geometry edge) were denoted as open porosity. Those pores that were open to the surface had their depths normalised with their height in the x-direction. The depths of all the internal pores were normalised by D_n and those closer than 1.5 diameters from the surface were identified. Two subsets of pores, those open to the surface and those close to the surface were thus identified (i.e. $< 1.5 \cdot D_n$). Linear interpolation was then used to fit the normalised depth to the stress concentration predicted by FEM (K_t (surface) in Figure 6).

Next, the distance between each pore centroid was calculated using an in-built MATLAB function. Again, the distance was normalised by D_n. Those pores with less than 3.5 diameters between their centroids were identified. Analysis of all the perimeter voxels in the two pores allowed the length of material between the pore edges to be calculated. Once more, linear interpolation was used to combine the theoretical FEM data regarding the effect of pores proximity to each other with the distance between the pores to approximate the increase in stress concentration due to other pores proximity (K_t (proximity) in Figure 7).

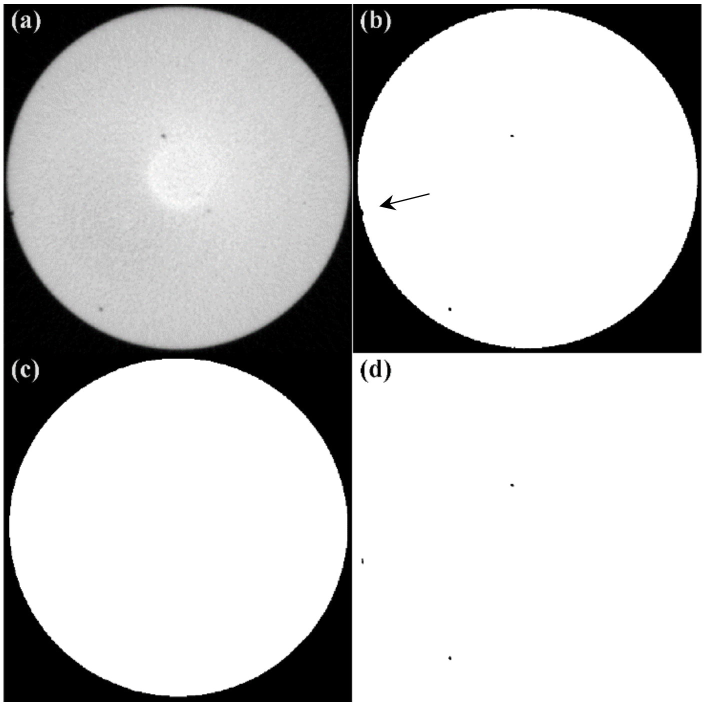

Figure S1. Identifying porosity from the CT Data. (a) slice of original data, (b) the segmented data (c) a convex hull used to approximate the ideal geometry and (d) by combining (b) and (c) it is possible to identify all the porosity including that which is open to the surface (arrowed).

Next, the distance between each pore centroid was calculated using an in-built MATLAB function. Again, the distance was normalised by D_n. Those pores with less than 3.5 diameters between their centroids were identified. Analysis of all the perimeter voxels in the two pores allowed the length of material between the pore edges to be calculated. Once more, linear interpolation was used to combine the theoretical FEM data regarding the effect of pores proximity to each other with the distance between the pores to approximate the increase in stress concentration due to other pores proximity (K_t (proximity) in Figure 7).

The final step was to estimate the stress at each pore location due to the sample geometry (Figure 8). To do this it was first necessary to convert the pore locations to the cylindrical coordinate system shown in Figure 8. This was achieved using the center and radii of the convex hull at each slice.

The stress at the pore location (σ_x) was then multiplied with any stress concentrations from the surface (K_t (surface)) or other pores proximity (K_t (proximity)) as well as the pore aspect ratio (K_t (AR)) to estimate the local stress around each pore. This was combined with the normal area (A_n) to provide an estimation of the relative stress intensity factor for each pore, as shown in equation (2).

After sorting the pores using equation (2) the pores with the highest relative stress intensity factors were identified. The binary slices defining each of the top 5 % most detrimental pores and their surrounding environment were then exported for further analysis. These slices included any neighboring pores and the sample surface, as originally defined by the CT data. The exported volume was centered on the pore and expanded by 5 times the pore size in each direction; hence larger pores resulted in a larger exported volume. From the modelling shown in Figures 6 and 7, it is clear that any other features, such as a free surface or other porosity, will have negligible influence if they are more than 5 diameters away from the pore of interest. Each pore location was also defined so that it could be located and checked against the fatigue cracks observed later, following testing.
